# Robust test method for time-course microarray experiments

**DOI:** 10.1186/1471-2105-11-391

**Published:** 2010-07-22

**Authors:** Insuk Sohn, Kouros Owzar, Stephen L George, Sujong Kim, Sin-Ho Jung

**Affiliations:** 1 Biostatistics and Bioinformatics Center, Samsung Cancer Research Institute, Samsung Medical Center, Seoul, 137-710, Republic of Korea; 2 Department of Biostatistics and Bioinformatics, Duke University Medical Center, NC 27710, USA; 3 CALGB Statistical Center, NC 27710, USA; 4 R&D Center, Komipharm International Co., LTD, Kyounggi-do 429-450, Republic of Korea

## Abstract

**Background:**

In a time-course microarray experiment, the expression level for each gene is observed across a number of time-points in order to characterize the temporal trajectories of the gene-expression profiles. For many of these experiments, the scientific aim is the identification of genes for which the trajectories depend on an experimental or phenotypic factor. There is an extensive recent body of literature on statistical methodology for addressing this analytical problem. Most of the existing methods are based on estimating the time-course trajectories using parametric or non-parametric mean regression methods. The sensitivity of these regression methods to outliers, an issue that is well documented in the statistical literature, should be of concern when analyzing microarray data.

**Results:**

In this paper, we propose a robust testing method for identifying genes whose expression time profiles depend on a factor. Furthermore, we propose a multiple testing procedure to adjust for multiplicity.

**Conclusions:**

Through an extensive simulation study, we will illustrate the performance of our method. Finally, we will report the results from applying our method to a case study and discussing potential extensions.

## Background

The objective of a time-course microarray experiment is to study the temporal dynamics of the expression profile for each gene. For many of these experiments, the primary objective is to identify genes for which these temporal profiles depend on a phenotypic, experimental or environmental factor. We mention three examples next. Wang and Kim [1] identify genes in *Caenorhabditis elegans *for which the expression level depends on the dauer state. Graham *et al*. [2] obtain RNA expressions from kidney tissue from patients ranging between 27 and 92 years old, to identify genes whose expression profiles are age dependent while adjusting for other phenotypic factors. Sekiguchi *et al*. [3] study the mRNA expression profiles of peripheral blood cells in patients with rheumatoid arthritis receiving a TNF-*α *inhibitor drug. Henceforth, for notational brevity we refer to these temporal expression profiles as gene time-trajectories or simply time-trajectories whenever understood from the context.

There is an extensive recent body of literature on statistical methodology for identifying genes whose time-trajectories depend on a factor. We provide a brief summary of representative works. Park *et al*. [4] propose a permutation-based two-way ANOVA model. Luna and Li [5] propose a statistical framework based on a shape-invariant additive error model utilizing periodically expressed guide genes. Storey *et al*. [6] estimate gene expression time-trajectories using splines and then approximate the null sampling distribution of the goodness of fit statistic using a bootstrap method. Sohn *et al*. [7] extend this method to carry out the inference using permutation resampling. Hong and Li [8] discuss a functional hierarchical model for detecting temporally differentially expressed genes between two experimental conditions for cross sectional designs, where the gene expression profiles are treated as functional data and modelled by basis function expansions. Finally, Angelini *et al*. [9] use a Bayesian hierarchical model along with Bayes factors for the inference.

**Figure 1:**
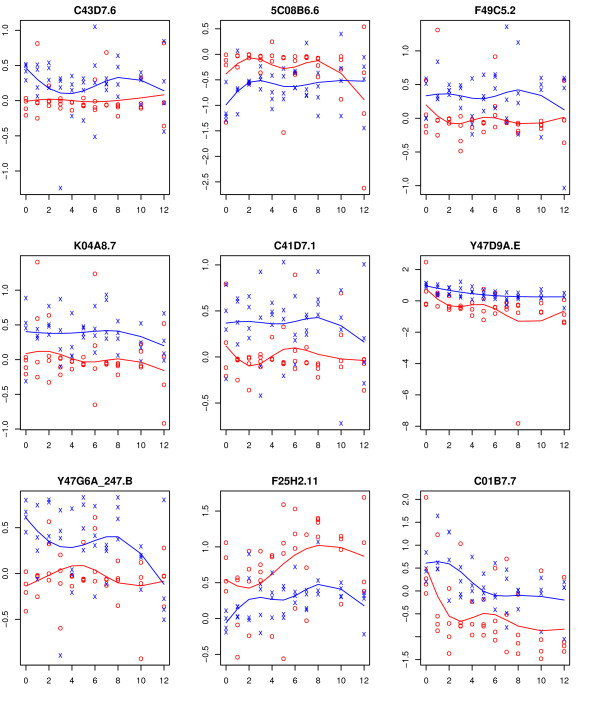
**The observations from control and experimental arms are represented by 'x' and 'o' respectively**. The fitted trajectory based on a natural spline basis of dimension four is superimposed for each group (blue for control group and red for experimental group).

One common concern with using the methods described in the above papers, which are based on estimating mean functions, is their sensitivity to outliers which is a common issue in most microarray, including time-course, experiments. Such outliers issue can be common in time-course microarray experiments. For example, our recent research [7] identified genes with potential outlier presence in the *Caenorhabditis elegans *dauer developmental data [1]. Figure 1 shows the observed and the fitted trajectory based on a natural spline basis of dimension four for nine genes with potential outlier presence. In this paper, we propose a robust testing method for identifying genes for which the gene-expression time-trajectories are different over time among *K ≥ *2 groups. The time-trajectories will be estimated using a quantile regression method. The discrepancy between the time-trajectories under the null hypothesis, where the time-trajectories for all *K *groups coincide, and the alternative, where the time-trajectory for some of the groups differ from the others, is quantified using an F-type goodness of fit statistic. Given that we are testing for a large number of genes, we will also propose a permutation-based multiple testing procedure to accurately control the family-wise error rate (FWER).

The remainder of this paper is organized as follows. In the next section, we provide a technical overview of the proposed method. Thereafter, we evaluate the procedure empirically using an extensive simulation study and by applying it to a published case study. We finalize the paper by a discussion of the empirical results and by considering extensions.

## Methods

We propose a robust method for identifying among *m *genes those whose expression time-trajectories depend on an experimental or phenotypic factor with say *K *≥ 2 groups or levels. We will assume that the expressions are observed at *L *distinct time-points, say 0 ≤ *t*_1 _< ... <*t*_*L *_and denote the number of observations for level *k *∈ {1, ..., *K*} of the factor at time *l *∈ {1, ..., *L*} by *n*_*kl*_. These time-points are assumed to be in common among the genes. Furthermore, we will assume that at least one observation is observed for each group at each time-point (i.e., *n*_*kl *_≥ 1). Let (*y*_*kli*1_, ..., *y*_*klim*_) denote the expression measurements for *m *genes at time *t*_*l*_(*l *= 1, ...,*L*) from subject *i*(= 1, ..., *n*_*kl*_) belonging to group *k*(= 1, ...,*K*). For a given gene *j*, we will assume that the expression profile for group *k *will follow a distribution whose conditional median at time *t *is *g*_*kj*_(*t*). For each gene *j*, following the discussions in [10] and [11], we will estimate each of the *K *conditional median functions by considering a class of quantile smoothing splines as solutions to

Where . Here  denotes the second derivative if *g*_*kj *_evaluated at *t *> 0. The parameter *λ *is the smoothing parameter. Here  denotes a class of "smooth" functions. Each function *g*_*kj *_is estimated based on the expressions belonging to group *k *for gene *j*. For gene *j *the hypotheses of interest are formulated as testing

Versus

Note that under the null, the function *g *will no longer depend on *k*. As such, under the null we will consider estimating a single time-trajectory for each gene as a solution to

The function *g*_*j *_is estimated based on all observations of gene *j *regardless of group membership by pooling within each time-point. Following [11] (appendix A.9), we will employ a pre-specified *p*-dimensional linear spline basis, say **W**(*t*_*l*_) = [*W*_1_(*t*_*l*_), ..., *Wp*(*t*_*l*_)], common to all *m *genes. The unknown parameters for gene *j *are denoted by *β*_*kj *_= [*β*_0,*kj*_, *β*_1,*kj*_, ..., *β*_*p,kj *_]. As discussed in [11] (see chapters 6 and 7) the estimation can be carried out efficiently using linear programming methods. Additional technical details for this optimization problem, including details about the family , are found in section 7.2.1 of [11]. The corresponding residual error sum for group *k *is then given by

where  are the corresponding estimators of the parameters. Under the null, the corresponding residual error is given by

We reject *H*_*j *_in favor of  for large values of the F-type statistic

We note that the principal objective is not to test the marginal hypotheses *H*_*j *_versus . Rather, we primarily aim to test the global null hypothesis  versus . To this end we will generate the null joint distribution of the test statistic  using a permutation resampling method. Under the ℍ_0 _, for all the genes, the observations within each time-points are exchangeable. As such, a permutation sample under the null can be obtained by permuting the observation within each time-point. Let *n_.l _*denote the number of observations at time *l*. The number of all possible permutations

is all but small sample-size cases prohibitively large. Therefore, we will approximate the exact sampling distribution using *B *random permutations. The FWER is defined by the probability of rejecting any null hypothesis *H*_*j *_under ℍ_0 _. We will employ a single-step multiple testing procedure controlling the FWER as described in [12] and [13]. The algorithm is summarized as follows.

1. Compute the the F-test statistics ) from the observed (non-permuted) data.

2. From the *b*-th (*b *= 1, ..., *B*) permutation sample compute a permutation replicate .

3. Single-step procedure to control the FWER:

(a) From the *b*-th permutation data, calculate .

(b) For gene *j*, calculate the adjusted p-value as .

(c) For a specified FWER level *α*, consider gene *j *significant if .

## Results

We investigate the performance of our method by conducting an extensive simulation study. This will be followed by a discussion of the application of our method to the *Caenorhabditis elegans *dauer developmental data discussed in [1]. These discussions will be limited to the two-sample case (i.e., *K *= 2). For notation brevity, we will refer to genes whose time-trajectory depends on the factor as prognostic and non-prognostic otherwise. Similarly, we will refer to the genes whose corresponding FWER *P*-value is less than the given nominal level as significant or non-significant otherwise. For all of these illustrations, as in [6], we will employ a B-spline basis with knots placed at the 0, 1/(*p *- 1),2/(*p *- 1), ..., (*p *- 2)/(*p *- 1), 1 quantiles of the observed time points pooled across both samples. For these illustration we set *p *= 4. Our method is developed within the framework of regression for estimation, and permutation resampling for the inference. For additional notational brevity we will adopt the acronym -PERM to refer to our method. To put the discussions in comparative perspective, alongside our results, we will provide those obtained by the permutation method of [7] and the bootstrap method by [6]. Given that these are regression methods, we will denote them by -PERM and -BOOT respectively. All of these analyses are carried out using the R statistical environment [14]. The function rq from the quantreg package [11] is used to estimate the quantile regression functions.

### Simulation Study

For the simulation study, the expressions will be generated from an outlier contaminated additive error model of the form

The first term, *μ*_*kj*_(*t*_*l*_), denotes the time-trajectory function at time *t*_*l *_for group *k*. For non-prognostic genes, we will set *μ*_1*j*_(*t*) = *μ*_2*j*_(*t*) = 0 while for prognostic genes, we will specify *μ*_1*j*_(*t*) = 0 and *μ*_2*j*_(*t*) = 1.5*e*^*-t *^respectively. The error terms are mutually independent and identically distributed according to a standard normal law. The second term in the model, *a*_*klij *_, represents the random outlier which will assume a value of 4, 0, or -4 with probabilities *π*/2, 1 - *π *and *π*/2 respectively. In the case of a normal law, the mean and median coincide. As such, in the absence of the outlier effect (i.e., *a*_*klij *_= 0 almost surely, or equivalent *π *= 0) the quantile function *g*_*kj*_(*t*) = *μ*_*kj*_(*t*) for all *t *> 0.

For these simulations, we will adopt a design similar to the *Caenorhabditis elegans *dauer developmental data, by choosing 11 time points *t *= 0, 1, 2, 3, 4, 5, 6, 7, 8, 10, and 12. We will generate four replicates at each time-point from each group (i.e., *n*_1*l*_= *n*_2*l *_= 4 for all *l*).

To evaluate the FWER, we generate *m *= 200 non-prognostic genes. A block exchangeable correlation structure with correlation coefficient *ρ*(= 0, 0.3 or 0.6) and block sizes of 10, is imposed on the measurement errors. The null distribution of the test statistic is approximated from B = 200 resampling (permutation or bootstrap) replicates. Empirical FWER is computed as the proportion of samples rejecting ℍ_0 _by our testing procedure at a two-sided FWER level of 0.05 among *N *= 200 simulations. Simulation results are reported in Table 1.

**Table 1:** Illustration of the empirical FWER based on a nominal two-sided 0.05 level, with ***m ***= 200 genes, ***N ***= 200 simulation samples and ***B ***= 200 resampling replications per sample. The outlier law realizes values -4,0 or 4 with probabilities *π*/2, 1 - *π *and *π*/2 respectively. Under simulation model 1, the outlier effect is added to all time-points. Under simulation model 2, the outlier effect is only added to the first and last time-points.

		Simulation 1			Simulation 2		
			
*π*	*ρ*	**-PERM**	**-PERM**	**-BOOT**	**-PERM**	**-PERM**	**-BOOT**
0	0	0.035	0.060	0.060			
	0.3	0.040	0.035	0.035			
	0.6	0.040	0.040	0.040			
0.05	0	0.040	0.040	0.040	0.065	0.075	0.155
	0.3	0.040	0.055	0.055	0.050	0.065	0.160
	0.6	0.060	0.045	0.055	0.075	0.055	0.130
0.1	0	0.035	0.040	0.050	0.035	0.040	0.355
	0.3	0.040	0.045	0.055	0.050	0.075	0.355
	0.6	0.060	0.045	0.045	0.050	0.055	0.295

As shown in Table 1, under the simulation model 1, where the outlier effects are identically distributed over the time-points, all three methods control the type I error rate. Under simulation model 2, where only the first and last time-points are contaminated by the outlier effect, the -BOOT method however fails to control the type I error rate. In this case, the type I error rate based on the -BOOT method is seemingly inflated by a factor of three when *π *= 0.05 or by a factor of six when *π *= 0.1. This can be explained by the fact that the parametric regression bootstrap is based on the assumption of homoscedasticity of the error terms.

Under simulation model 1, the error terms as the sum of the outlier and measurement error components, although no longer normally distributed, are identically distributed within and among all time-points and groups. Under simulation model 2, the error terms are no longer homoscedastic. As such, it is not surprising that the -BOOT method may not be adequate in this situation.

To investigate the global power (i.e., the probability of rejecting at least one null hypothesis) of this procedure, we generate 10 prognostic genes and 190 non-prognostic genes. A correlation structure similar to that of the FWER case is specified. The corresponding results are reported in Table 2.

As illustrated in Table 2, in the absence of an outlier effect, the power for our method is slightly lower than the other two methods. As this effect becomes more pronounced, our method gains an advantage with respect to power. A similar trend is observed under simulation model 2. One should note that the power listed for -BOOT under this scenario is not the power at the two-sided FWER level of 0.05, but rather the power at the inflated type I error rate as observed in Table 1. As such, it is erroneous to conclude that that the -BOOT method is more powerful.

**Table 2:** Illustration of the empirical Power based on a nominal two-sided 0.05 FWER level, with ***m ***= 200 genes, ***N ***= 200 simulation samples and ***B ***= 200 resampling replications per sample. The outlier law realizes values -4,0 or 4 with probabilities *π*/2, 1 - *π *and *π*/2 respectively. Under simulation model 1, the outlier effect is added to all time-points. Under simulation model 2, the outlier effect is only added to the first and last time-points.

		Simulation 1			Simulation 2		
			
*π*	*ρ*	**-PERM**	**-PERM**	**-BOOT**	**-PERM**	**-PERM**	**-BOOT**
0	0	0.960	0.995	0.995			
	0.3	0.880	0.940	0.925			
	0.6	0.750	0.855	0.845			
0.05	0	0.910	0.800	0.790	0.890	0.880	0.970
	0.3	0.835	0.755	0.755	0.845	0.825	0.900
	0.6	0.680	0.630	0.605	0.835	0.805	0.935
0.1	0	0.760	0.485	0.480	0.895	0.740	0.930
	0.3	0.675	0.415	0.425	0.660	0.585	0.885
	0.6	0.650	0.410	0.370	0.730	0.530	0.895

we evaluated the empirical power and FWER under the simulation model 1 and 2 for *π *= 2. The result of *π *= 2 have a similar trend the results of *π *= 0.05 and *π *= 0.1 under simulation model 1 and 2. We also evaluated the different proportions for 20 prognostic genes (10%) and 180 non-prognostic genes (90%) and 5 prognostic genes (2.5%) and 195 non-prognostic genes (97.5%). These results have a similar trend the result of 10 prognostic genes (5%) and 190 non-prognostic genes (95%).

### Case Study

In this section, we will summarize the results from applying our proposed method to the *Caenorhabditis elegans *dauer data. Wang and Kim [1] use cDNA microarrays to profile gene expression time-trajectories during the transition from the dauer state to the non-dauer state (experimental group) and after feeding of starved first laval stage worms (control group). The cDNA microarray expressions were measured on *m *= 18, 556 genes. For the experimental group, the worms are harvested at 0, 1.5, 2, 3, 4, 5, 6, 7, 8, 10, and 12 hours after feeding with three to four replicates at each time-point. For the control group, are harvested 0, 1, 2, 3, 4, 5, 6, 7, 8, 10, and 12 hours after feeding withe four replicates at each time-point. For this illustration, we have set the *t *= 1.5 time-point in the experimental group to *t *= 1. This data set is available for download from http://cmgm.stanford.edu/~kimlab/dauer/.

**Figure 2:**
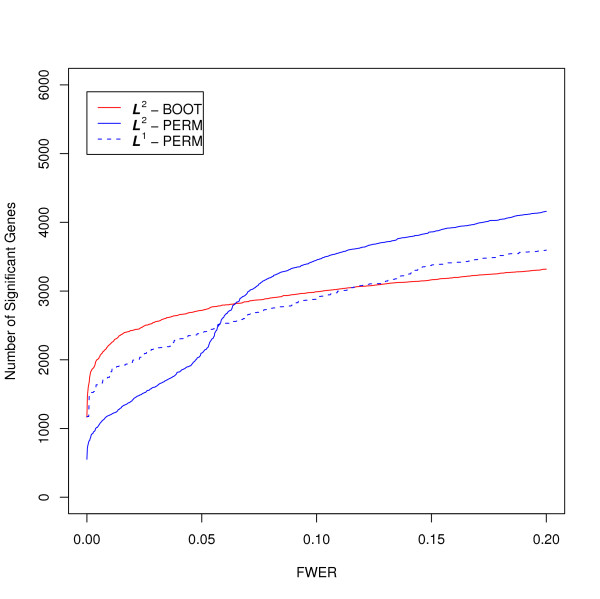
**The number of significant genes at a given FWER level (from 0 to 0.2) based on the -PERM, -BOOT and -PERM adjusted *P*-values**.

For the case study based on the Wang and Kim [1] data, the number of significant genes, identified at a given FWER ∈ (0, 0.2] based on each of the three methods, is shown in Figure 2. We note that for smaller FWER levels (less than 0.05), the -BOOT method identifies the largest while the -PERM method identifies the smallest number of significant genes. This is reversed for larger FWER levels. The number of significant genes identified by the -PERM method is consistently between the numbers identified by the other two methods. The set of significant genes identified by -PERM is, however, not a proper subset of genes identified by methods. In other words, the -PERM method is identifying potentially novel genes missed by the other two methods.

**Figure 3:**
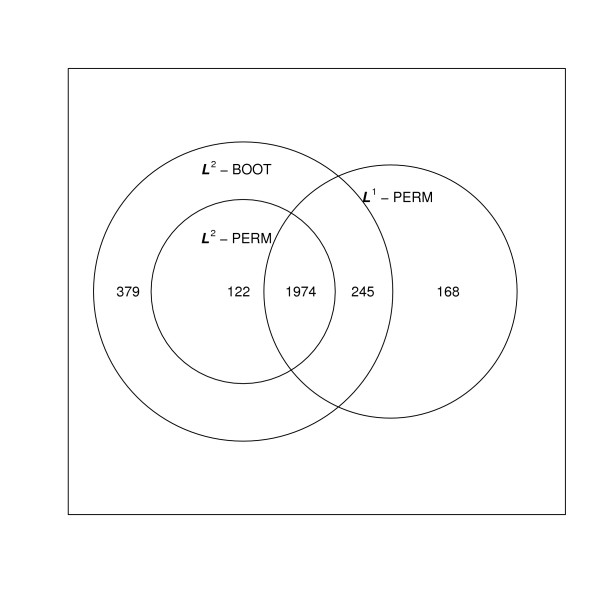
**Venn diagrams for the number of significant genes at the 0.05 FWER level based on the -PERM, -BOOT and -PERM adjusted *P*-values**.

**Figure 4:**
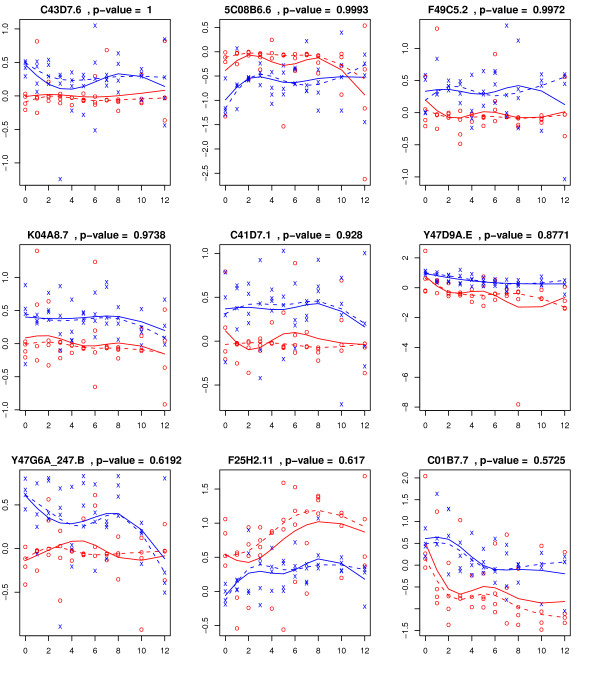
**Observed expressions and estimated expression trajectories for the top four genes, from the **[1]**data, among the 168 genes identified by -PERM but neither by -PERM nor by -BOOT, at the 0.05 FWER level, are shown here**. For each of these 168 genes, we calculate the absolute difference between the -PERM and -PERM FWER *P*-values. The top nine genes ranked according to the magnitude of these differences are selected. The observations from the control and experimental arms are represented by 'x' (blue) and 'o' (red) respectively while the group-wise quantile and mean regression curves are denoted by dashed and solid lines respectively. The adjusted P-value by ****-PERM is provided for each gene.

**Figure 5:**
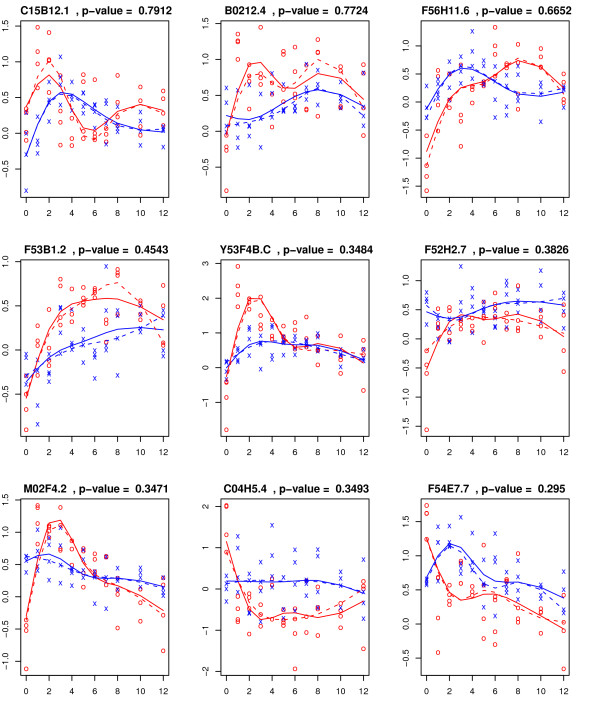
**Observed expressions and estimated expression trajectories for the top four genes, from the **[1]**data, among the 379 genes identified by -BOOT but neither by -PERM nor by -PERM, at the 0.05 FWER level, are shown here**. For each of these 379 genes, we calculate the absolute difference between the -PERM and -BOOT FWER *P*-values. The top nine genes ranked according to the magnitude of these differences are selected. The observations from the control and experimental arms are represented by 'x' (blue) and 'o' (red) respectively while the group-wise quantile and mean regression curves are denoted by dashed and solid lines respectively. The adjusted P-value by -PERM is provided for each gene.

We provide a Venn diagram on the set of of genes identified as significant at the 0.05 FWER level in Figure 3. There are 1974 significant genes in common among the three methods. The -PERM identifies 168 genes not identified by the other two methods. We rank these 168 genes according to the difference of their respective *P*-values from the -PERM and -PERM methods and show the top nine genes according to this ranking in Figure 4. As illustrated in the simulation study, the mean model by regression is not robust to the outliers. There are 379 genes identified as significant by the -BOOT method only. The top nine genes, based on the magnitude of the differences of the corresponding 379 -BOOT and -PERM FWER *P*-values, are shown in Figure 5. As illustrated in the simulation study, -BOOT may be severely anti-conservative if the error terms are heterogeneous over time [7]. The supplementary material provides the biological properties of 40 genes (out of 168) that are identified only by -PERM [see Additional file 1].

## Discussion

In these discussions, we have assumed that any difference, including vertical shifts, among the time trajectories, are biologically relevant and of interest. In some applications, one may want to ignore vertical shifts, as these may be often caused by batch effects, and primarily focus on genes for which there are actual differences among the time trends. The procedures we have discussed can be easily modified to accommodate this situation. To this end, for gene , the *K *sample means for each group are computed. The algorithm is then applied to the centered versions of the observed expressions . Although the illustration presented in this paper have been limited to the two-sample case (*K *= 2), as shown in the methods section, the method can be extended to the case where *K *> 2. The method can be easily extended to account for multiplicity by controlling a false-discovery rate (FDR). The unadjusted permutation *P*-value for gene *j*, based on the notation in the algorithm presented in the methods section, is . FDR adjusted *P*-values can then be computed based on these unadjusted *P*-values. We finally note that the method can also be applied in the one-sample cases. In this setting, one is interested in identifying genes whose time-trajectories are time-dependent. The marginal hypotheses are formulated as testing *H*_*j *_: *g*_*j*_(*t*) = *c*_*j *_for all *t *> 0 versus *H*_*j *_: *g*_*j*_(*t*) ≠ *c*_*j *_for some *t *> 0 for a constant say *c*_*j *_. As under the null, all of the expressions are exchangeable, the null sampling distribution is generated by permuting all observed expressions for a given gene. The corresponding null residual error is obtained as  where  is the sample mean for the *n *expressions observed for gene *j*. For many regressions problems, the target function to be estimated is the mean of the distribution of the outcome conditional on a set of co-variables. In a time-course microarray experiment, this would correspond to the mean of the expression profile over time. In this paper, we have proposed to estimate the conditional quantile, rather than the conditional mean, of the distribution of the outcome variable a as function of time. Specifically, we use the special case of the median. Consider the standard additive mean regression problem of the form *Y*_*i *_= *g*(*t*_*i*_) + *ϵ*_*i*_, where *g*(*t*) is the conditional mean of *Y *at time *t *and *ϵ *is a mean-zero error term. One criterion that is often used to find an estimate of *g *is to minimize Σ*_i_*(*Y*_*i *_- *g*(*t*_*i*_))^2^. Restricting this optimization to the set of linear functions yields the standard least-squares estimate. Optimizing over the set of all "smooth" functions yields an estimator that interpolates the observations. As a balancing act between these two extremes, one may consider optimizing the following criterion

where ∫(*g''*(*t*))^2^*dt *is a so called penalty term. The amount of smoothing is determined by the parameter *λ ∈ *(0, ∞). The estimation procedure used in this paper is based on a similar regularization approach where the terms (*y*_*i *_- *g*(*t*_*i*_))^2 ^are replaced by *ρ*(*y*_*i *_- *g*(*t*_*i*_)) and the penalty term ∫(*g''*(*t*))^2^*dt *is replaced ∫|*g''*(*t*)|*dt*.

## Conclusion

We proposed a robust method for identifying genes whose time trajectories depend on a phenotypic or experimental factor. Furthermore, we proposed a multiple testing procedure to adjust for multiplicity. Our method is based on regression type estimator. Through an extensive simulation study, we observed our method accurately control the FWER and the mean model by regression is not robust to the outliers.

## Authors' contributions

IS proposed the research project. IS and KO performed statistical analysis and wrote the manuscript. SLG and SHJ contributd to the research and critically revised the manuscript. SK conducted the biological interpretation of the statistical analysis results. All authors read and approved the final manuscript.

## Supplementary Material

Additional file 1**Properties of 40 genes that are discovered only by -PERM**. The data provided the biological properties of 40 genes that are discovered only by -PERM.Click here for file
